# Insertion of the voltage-sensitive domain into circularly permuted red fluorescent protein as a design for genetically encoded voltage sensor

**DOI:** 10.1371/journal.pone.0184225

**Published:** 2017-09-01

**Authors:** Liubov A. Kost, Evgeny S. Nikitin, Violetta O. Ivanova, Uhna Sung, Ekaterina V. Putintseva, Dmitry M. Chudakov, Pavel M. Balaban, Konstantin A. Lukyanov, Alexey M. Bogdanov

**Affiliations:** 1 Shemyakin-Ovchinnikov Institute of Bioorganic Chemistry, Moscow, Russian Federation; 2 Institute of Higher Nervous Activity and Neurophysiology, Moscow, Russian Federation; 3 Center for Functional Connectomics, Korea Institute of Science & Technology, Seoul, Korea; 4 Central European Institute of Technology, Masaryk University, Brno, Czech Republic; 5 Pirogov Russian National Research Medical University, Moscow, Russia; 6 Nizhny Novgorod State Medical Academy, Nizhny Novgorod, Russia; University of Cambridge, UNITED KINGDOM

## Abstract

Visualization of electrical activity in living cells represents an important challenge in context of basic neurophysiological studies. Here we report a new voltage sensitive fluorescent indicator which response could be detected by fluorescence monitoring in a single red channel. To the best of our knowledge, this is the first fluorescent protein-based voltage sensor which uses insertion-into-circular permutant topology to provide an efficient interaction between sensitive and reporter domains. Its fluorescent core originates from red fluorescent protein (FP) FusionRed, which has optimal spectral characteristics to be used in whole body imaging techniques. Indicators using the same domain topology could become a new perspective for the FP-based voltage sensors that are traditionally based on Förster resonance energy transfer (FRET).

## Introduction

A lot of different techniques and approaches for simultaneous recording of the electrical activity from a number of nerve cells are currently applied for understanding important processes occurring in the brain at the neuronal circuits level. Voltage sensitive fluorescent biosensors are among the most successful approaches in this area. Such indicators of membrane potential are typically represented by the relatively small proteins, coding sequences of which can be introduced to the genome of the organism or transiently expressed in the cells of interest. This approach allows observing voltage changes with minimal invasivity during long term experiments. The sensor gene can be introduced to the genome under a control of a specific promoter and thereby expressed in a predefined tissue or in a cell specific way.

Biosensors of this type are subjects for continuous improvements and optimization. An ideal sensor should have high spatial and temporal resolution, as well as a high contrast in spectral responses to the changes of membrane potential. Other critical characteristics are wide range of detectable voltages, minimal response time and low toxicity *in vivo*. Looking forward to a routine experimentation using the FP-based biosensors, one should also pay attention to such problem as detection simplicity and reliability. It is reasonable to assume that the bright sensor emitting in single channel is the most preferable solution for the majority of experimental situations. Moreover, *in vivo* studies require an enhanced penetrating capacity of emitted light, which is characteristic for the red-to-infrared wavelength range [1]. Signal of the perfect biosensor should be reliably recorded from the body surface of the whole intact organism. The best of currently available biosensors still didn't reach the perfect combination of all these parameters [2,3].

Basically, the molecule of biosensor consists of two parts. Voltage sensitive domain (VSD) is a transmembrane protein commonly originating from different voltage-sensitive ion channels [4–7]. VSD is linked to reporter domain that is usually represented by fluorescent proteins (FPs) and their modifications. It can be a single FP (including circularly permuted variants, cpFPs) [4,8,9], or a pair of FPs interacting with each other as a FRET-pair [7,10–13]. Both sensitive and reporter components determine the entire performance of the particular sensor. Single FP by itself acts as a reporter domain in several biosensors of membrane potential [4,14] but the contrast of such type of sensors is quite low due to the high stability of the FP beta-barrel, which protects a chromophore group. Circularly permuted FPs, which possess new N- and C-termini in a close proximity to the chromophore and are therefore considered as the quick response reporters were also previously used for voltage sensors engineering [8]. Performance of such sensors, however, was significantly lower compared to their FRET counterparts, probably, due to non-optimal topology of the polypeptide chain. Namely, voltage-sensitive domain was fused to the N-terminus of cpmKate, thus utilizing the single interaction point between sensitive and reporter parts of the constructs. At the same time, at least 6 topologies potentially applicable for sensors design were described in literature [15]. One should mention that the efficiency of the particular sensor topology depends on a plenty of factors including a mechanism of a sensitive domain functioning, and generally cannot be predicted *a priori*.

Here we aimed to modify the reporter domain of the VSFP-Butterfly1.2 voltage sensor [12] in a way to simplify detection of its signal and shift detection to a red part of the spectrum. Originally, reporter part of this sensor was built with two FPs (yellow mCitrine and red mKate) forming a FRET-pair. These FPs were attached to the N- and C-termini of a sensitive domain (VSD) which had been made as a chimera of voltage-activated potassium channel Kv3.1 and *Ciona intestinalis* voltage-sensitive phosphatase (Ci-VSP) [16]. Conformational changes in VSD produced by membrane voltage shifts lead to the FRET efficiency alterations that can be detected using a fluorescence microscopy. We assumed that the voltage-sensitive core of VSFP-Butterfly1.2 is quite promising for novel sensors, and that’s why we chose it for the current project.

## Materials and methods

### Cell lines

Human embryonic kidney HEK293 [17] and Rat pheochromocytoma PC12 [18] cell lines were from the Institute of Bioorganic Chemistry collection of cell lines. PC12 cells successfully passed the test of NGF-induced neuronal differentiation. Lines were not additionally tested for identity and contamination as it was used for characterization of fluorescence proteins rather than specific cell biology questions. For cell culture and transfection procedure protocols see S1 Appendix.

### Permutation

To obtain permutants we amplified the fragments of tandem-FusionRed (S1 Fig) with different pairs of internal primers annealing to the respective permutation points (forward primer annealed to the first copy of FP, reverse primer–to the second one). Then PCR-products were cloned into pQE30 expression vector (Qiagen) using BamHI/HindIII restriction endonucleases. All variants were expressed in *E*.*coli* strain XL1 Blue.

### Split

We used a two-promoter vector system pAqMHalvesZip1 (kindly provided by Dr. Dmitry Shcherbo) to construct a plasmid with split fragments of FusionRed fused to the leucine zippers. Each of two FP-zipper fusions was cloned after two individual CMV promoters. To make two different versions of split FusionRed we separately amplified FRpart^1-73^ and FRpart^76-233^ as well as FRpart^1-188^ and FRpart^189-233^ fragments and inserted these pairs into pAqMHalvesZip1 using HindIII/NotI sites for the first part of the sequence and AgeI/EcoRI for the second one, respectively. The final plasmids (pZip189-188 and pZip76-73, see cloning scheme in S2 Fig) were used for HEK293 cells transient transfection.

### VSD insertion into cpFR

Voltage sensitive domain was PCR-amplified using pCAG-VSFP Butterfly 1.2 vector (kindly provided by Dr. Thomas Knöpfel) as a template. Split cpFusionRed fragments were attached to N- and C-termini of VSD using overlap-extension PCR. Resulting PCR fragments (FRpart^189-233^-VSD-FRpart^1-188^ and FRpart^76-233^-VSD-FRpart^1-73^) were then subcloned into pEGFP-N1 (Clontech) vector with AgeI/NotI restriction sites and named pVSD-FR^189-188^ and pVSD-FR^76-73^ (Cloning scheme in S3 Fig).

### Electrophysiology recordings and imaging of PC12 cells

A coverslip with transfected PC12 cells was placed in the recording chamber of an upright fluorescence microscope and superfused with bath solution (in mM: 125 NaCl, 25 NaHCO_3_, 27.5 glucose, 2.5 KCl, 1.25 NaH_2_PO_4_, 2 CaCl_2_ and 1.5 MgCl_2_, pH 7.4) pre-aerated with 95% O_2_, 5% CO_2_. Whole cell recordings were performed in voltage-clamp mode. Borosilicate glass electrodes (resistance of 5 MΩ) were filled with a solution containing (in mM) 132 K-Gluconate, 20 KCl, 4 Mg-ATP, 0.3 Na_2_GTP, 10 Na-Phosphocreatine, 10 HEPES, pH 7.25 (all from Sigma, St. Louis, MO, USA). Voltage steps were applied in whole-cell voltage clamp mode with an Axoclamp 2B amplifier (Axon Instruments, USA) driven with a DigiData 1440A ADC board (both from Molecular Devices, Sunnyvale, CA, USA). For precise positioning of the patch pipette, the rig was equipped with a motorized micromanipulator Junior (Luigs and Neumann, Germany) mounted on an air table. During recording the coverglasses were kept at RT (22 ± 2°C) and perfused at a constant rate of 3 ml/min. Confocal imaging of signals induced by voltage steps was performed with a Zeiss LSM 5 Live microscope (Germany) in epifluorescent mode (excitation 532 nm, emission 550LP) at 500 fps rate. The measured optical signal reflected the change in fluorescence/light emission relative to its mean value (DF/F). Signals from each cell were analyzed as step-triggered average of 15–20 trials.

### Electrophysiology recordings of HEK293 cells

Electrophysiological recordings from HEK293 cells at 24 hours post-transfection were performed in a perfusion chamber with the bath temperature kept at 33°C by a temperature controller. The bathing solution was KRH (Krebs-Ringers HEPES) solution (120 mM NaCl, 4.7 mM KCl, 1.2 mM KH_2_PO_4_, 1.2 mM MgSO_4_, 10 mM HEPES, 2.2 mM CaCl_2_, and 1.8 mg/ ml D-glucose, pH 7.4). We used 3–5 MΩ glass patch pipettes (capillary tubing with 1.5/0.75 mm OD/ID from World Precision Instruments, FL) that were pulled on a P-97 Flaming/ Brown type micropipette puller (Sutter Instrument Company, CA). The pipette solution contained 120 mM K-aspartate, 4 mM NaCl, 4 mM MgCl_2_, 1mM CaCl_2_, 10 mM EGTA, 3 mM Na_2_ATP and 5 mM HEPES, pH 7.2. Voltage-clamp recordings in the whole-cell configuration were performed using a Patch Clamp PC-505B amplifier (Warner Instruments, CT). We stepped the membrane potential from a holding potential at -70 mV to -170 mV, -20 mV, +30 mV. Whole-cell patch clamped HEK293 on a 0.08–0.13 mm thick cover slip were imaged on an Olympus IX71 inverted microscope (Olympus, Japan) using an Olympus UPLANSAPO 60x/1.35 NA oil immersion objective and a XBP 75 W/2 OFR Xenon short arc lamp (OSRAM, MI) with a stabilized power supply (Cairn Research, UK). Illumination intensity was 2.5 mW mm^-2^. We used a 562/40 nm excitation filter, a 593 nm dichroic mirror, and 641/75 nm emission filters (Semrock, NY). The fluorescence images were demagnified by an Optem^®^ zoom system A45699 (Qioptiq LINOS Inc. NY) and projected onto a FastCMOS-128x camera (RedShirtImaging) controlled by NeuroPlex software (RedShirtImaging). The images were recorded at a frame rate of 500 fps with the FastCMOS-128x camera[19,20].

## Results and discussion

In order to develop a new design of voltage sensors, we decided to use insertion-into-cpFP topology as a basis for interdomain communication. One can suppose that among several well-described protein topologies used earlier in molecular sensors, this design deserves special interest. Here, unlike in typical cpFP-based sensor, FP polypeptide chain is split into two fragments by VSD, and new FP termini are not fused to the sensitive domain but free to interact with each other to form a mature RFP. This principle looks similar to splitFP re-association used in bimolecular fluorescence complementation (BiFC) technique though in this case FP parts joining happens intramolecularly. Considering that VSD undergoes large conformational shifts significantly changing relative spatial positions of its termini, we assumed that sensor topology which involves both ends of the sensitive domain to interaction might be more favorable than ‘classic’ cpFP-VSD fusion.

Red fluorescent protein (RFP) was chosen as a template for the reporter part construction. As all natural RFPs are oligomers, we used a recently developed monomeric RFP FusionRed, which was shown to be a good partner for fusion constructs, and possesses low toxicity and high pH- and photo-stability [21].

### Circular permutants engineering

As a first step, we tested several circularly permuted variants of FusionRed. We analyzed the crystal structure of mKate, a predecessor of FusionRed (PDB ID: 3BXA [22]), and chose a set of reasonable points for permutation. Using the tandem construct [23] of FusionRed we engineered 23 variants of circularly permuted FusionRed (cpFR). One group of them had a polypeptide chain break in loop regions at positions (numbering by mKate): 75–74, 76–73, 87–85, 150–151, 152–151, 167–166, 168–167, 169–168, 189–188, 167–167, 167–168, 168–168; and the second group had a break in beta-strands: 142–141, 143–142, 144–143, 145–144, 142–142, 142–143, 142–144, 143–143, 143–144, 144–144, 150–149. Spectral properties of all permutants were characterized (Table 1). We chose two fast–maturing variants with sufficient fluorescence brightness, namely 76–73 and 189–188, for further engineering of split FusionRed.

**Table 1:** Relative brightness and maturation speed of FusionRed permutants.

cpFusionRed breaking point	Colony brightness 48 h at 37°C (% of Fusion Red)	Extinction coefficient (M^-1^ cm^-1^)	Quantum yield	Brightness of protein (% of FusionRed)	Maturation speed 24 h
**75–74**	53,5	83000	0,04	18,3	-
76–73 R126I*	**47,3**	**76000**	**0,10**	**41,9**	**+++**
**87–85**	48,6	130000	0,11	78,8	+
**150–151**	74,7	124500	0,16	110	+
**152–151**	110,6	148000	0,14	114	+
**167–166**	94,5	106000	0,16	93,5	+
**167–167**	61,3	117000	0,17	109,6	-
**167–168**	59,1	125000	0,15	103	+
**168–168**	62,5	119000	0,17	111,5	+
**168–167**	105,0	97000	0,13	69,5	+
**169–168**	113,5	81000	0,14	62,5	+
**189–188**	**133,1**	**109000**	**0,14**	**84,1**	**++**
**FusionRed**	100,0	95500	0,19	100,0	++++

Red fluorescence quantum yield at 574 nm excitation were measured using FusionRed as the reference standard. Extinction coefficients were measured by alkali-denatured chromophore method (see S1 Appendix).

Relative brightness of chromophore (extinction coefficient *quantum yield) was compared with FusionRed (100%). Maturation speed was measured by comparing *E*.*coli* colony brightness in 24 hours incubation at 37°C using Adobe Photoshop CS5 software. "-"—shows the absence of detectable fluorescent signal after 24 hours of incubation at 37°C, "+++"—high fluorescence level, "++"—medium fluorescence level and "+" is a low—fluorescence level. cpFR variants which are not shown in this table didn't have detectable fluorescent signal after 48 hours at 37°C.

*—cpFR76-73 R126I is an optimized variant of cpFR76-73, which has improved brightness and maturation speed due to the substitution R126I introduced during the random PCR-mutagenesis.

We hypothesized that properties of circularly permuted protein would highly correlate with that of the reconstituted splitFP “broken” at the same point. Taking into account this assumption and the intention to use permutants with split polypeptide chains as fluorescent cores for molecular sensors, we based the choice of permutants on two criteria. Firstly, fast maturation and relatively high brightness, secondly, diversity of chosen breakpoints in respect of protein secondary structure topology and chromophore proximity. On the other hand, we attempted to design split RFPs consisting of strongly unequal parts, smaller of which could be conformationally flexible providing good sensor response. Thus, we chose two fast–maturing modifications with sufficient fluorescence brightness, namely 76–73 and 189–188, for further engineering of split FusionRed. In case of 76–73, the smaller part was N-terminal fragment of FusionRed, and for 189–188, conversely, the C-terminal fragment.

We carried out further optimization of chosen permutants. Optimization included a round of random PCR mutagenesis that introduced the substitution R126I into cpFR76-73 (numbering by FusionRed). Mutants selection was performed using improved brightness and maturation speed criteria. cpFR76-73 and cpFR189-188 chromophores maturation speed was shown to be close to that of FusionRed, and fluorescence intensities compared to that of FusionRed were 47% and 65%, respectively (Table 1).

According to the fluorimetric data, the proteins FusionRed and cpFusionRed had virtually identical fluorescence excitation and emission spectra (not shown). The excitation maximum was at 574 nm, and the fluorescence emission maximum at 610 nm. The fluorescence quantum yield, molar extinction coefficient and the brightness of obtained cpFRs are shown in Table 1.

### Split FusionRed engineering and testing

**Fig 1:**
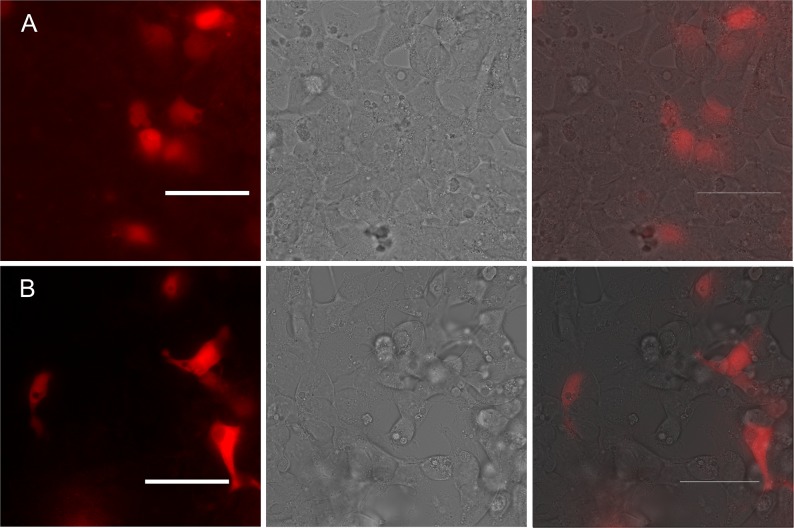
HEK293T cells transiently transfected with (A) pZip76-73 and (B) pZip189-188. Left panel—fluorescent images, central panel—respective images in transmitted light, right panel–overlays. Scale bar 50 μm.

Leucine zipper is a popular model system for engineering and testing split FPs. This model is typically used for testing protein-protein interactions research techniques, particularly, bimolecular fluorescent complementation [24]. Leucine zipper represents a common three-dimensional structural motif [24–26] which acts as a dimerization unit *in vivo* as well as *in cellulo* and *in vitro* model systems. We constructed eukaryotic expression vector with two pairs of split parts of FusionRed and leucine zippers (NZ or CZ), with each zipper fused to a part of FP under control of the same promoter (S2 Fig). Both split variants (FR189-188-zip and FR76-73-zip) displayed red fluorescence signal when expressed in mammalian cell line HEK293T (Fig 1). Brightness of FR189-188-zip was similar to that of the parental FusionRed. The second variant, split FR76-73-zip, was about 5-fold dimmer than FusionRed. Thus, we demonstrated that both split RFPs can successfully re-associate and form a mature fluorescent protein in mammalian cells. Importantly, we did not observe spontaneous association of split fragments without zippers under the same expression conditions (data not shown). Split RFPs themselves are potentially useful tools for different tasks connected with a detection of protein-protein interactions and dimerization, visualization of protein aggregation, folding, topology, conformational changes, multiple protein complexes formation [24].

### Construction of voltage sensor

**Fig 2:**
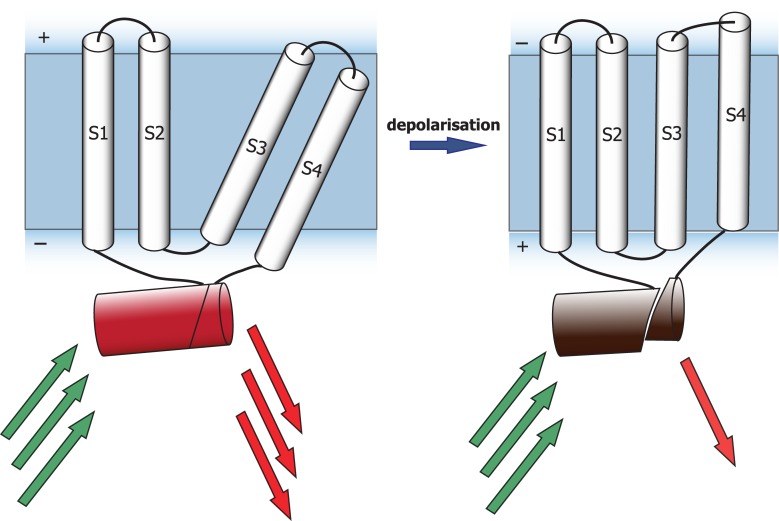
Schematic illustration of a sensor design principle. Depolarization causes a movement in transmembrane voltage sensitive domain, which results in a change of FP conformation and then a decrease of fluorescent signal intensity. S1-S4—are transmembrane voltage sensitive domains of the Butterfly 1.2 [7], the red barrel fused to N- and C-termini of VSD is a red FP FusionRed split at the points 189–188 or 76–73.

The concept of biosensor based on insertion-into-cpFP topology was described above and is shown in the Fig 2. Two nonfluorescent portions of red FP FusionRed were linked to the N- and C-termini of voltage sensitive domain (VSD) in a way to provide free interaction of regions, which formed polypeptide chain in the original fluorescent protein.

VSD is a chimerical transmembrane voltage sensitive domain of Butterfly 1.2 sensor, originally constructed in Thomas Knöpfel's lab [12], and proved itself as the perspective sensitive core for voltage biosensors due to fast kinetics and reliable membrane targeting in different cell types. Response to membrane potential changes is provided by a significant conformational shift of the fourth transmembrane helix (S4) which could be effectively transmitted to a fluorescent domain. In our design, the conformational movements occurring in Butterfly1.2 due to the changes of membrane potential would affect the reconstituted FusionRed fluorescence by a mechanical stretching of the non-covalently assembled beta-barrel. Recording of fluorescent response in this case would be performed in a single red channel as a simple change of signal intensity. We engineered two variants of VSD-split FusionRed constructs, pVSD-FR76-73 and pVSD-FR189-188 (S3 Fig).

### cpFusionRed-based voltage sensor eukaryotic expression and testing

We transiently expressed both constructs in PC12 cells and tested their voltage-sensitivity after cells differentiation with nerve growth factor (NGF) using patch-clamp technique in a whole cell configuration.

PC12 cells originate from a chromaffin tumor of the rat adrenal medulla which is derived from the neural crest of embryo [18]. PC12 could be either held in stem cells-like state, characterized in suspension cultivation and active proliferation, or differentiated into the neuron-like cells which attach to the substrate and form a dendrite-like structures. Differentiation is typically induced with the NGF treatment. Though differentiated PC12 are not considered adult neurons, they nevertheless represent a popular model system for neurophysiology combining neuron-like phenotype with relatively simple cultivation protocols.

**Fig 3:**
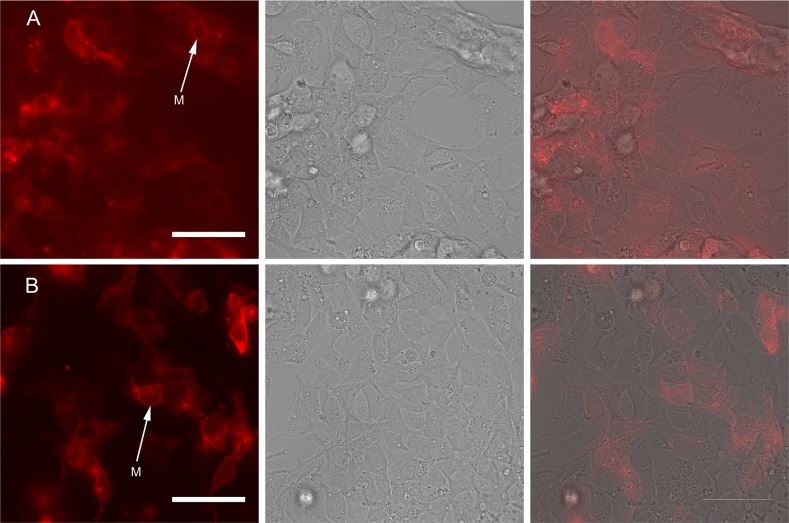
HEK293T cells transiently transfected with (A) pVSD-FR76-73 and (B) pVSD-FR189-188. Left panel—fluorescent images, central panel—respective images in transmitted light, right panel—overlays. Scale bar 50 μm. Arrows with “M” letter show plasma membranes. Predominantly membrane localization of proteins and a few aggregates in the cytoplasm can be observed.

Also we performed a series of similar experiments using another cell line HEK293. Cells were transiently transfected with pVSD-FR189-188 and pVSD-FR76-73 (Fig 3) and patched during simultaneous recording of fluorescence.

For pVSD-FR76-73 construct we observed aggregation of the protein in the perinuclear space and a relatively weak signal at the plasma membrane after the expression in the both model systems (HEK293 and PC12). Dim fluorescent signal and low expression level in combination with the membrane localization problems lead to the inability to record a significant shift of the fluorescent signal in response to the changes of membrane potential.

**Fig 4:**
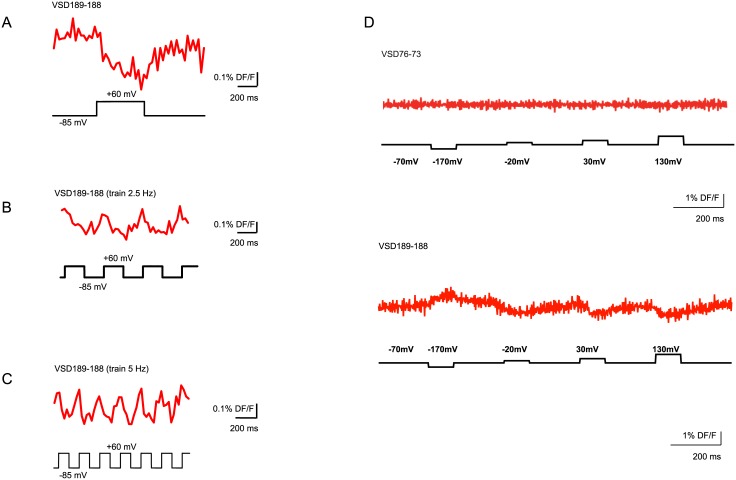
Functional characterization of cpFR-based voltage VSD variants in a voltage-clamped PC12 (A-C) and HEK293 (D) cells. The fluorescence of probe VSD-FR189-188 is sensitive to the transmembrane potential changes. (A-C) Fluorescence changes (top traces) to single voltage steps (A) and trains of 2.5Hz (B) and 5Hz (C) were recorded with the confocal microscope. Step commands are shown below each trace. (D) Comparison of VSD-FR189-188 signal (bottom trace) induced by a command protocol of a single hyperpolarizing step and the three subsequent incrementing depolarizing steps to the recording of VSD-FR76-73 made with the same protocol (top trace) is shown. The images were recorded at a frame rate of 500 fps, averaged traces of 16 trials, and shown without filtering.

In contrast, VSD-FR189-188 had an appropriate membrane localization and a detectable decrease of fluorescence intensity in the red channel in response to changes in membrane potential. To characterize voltage-sensitive properties of the prospective voltage probe VSD-FR189-188, we applied voltage steps to voltage-clamped PC12 cells transfected with VSD189-188 and recorded the fluorescent changes with a fast confocal microscope. A voltage step from the holding potential of -85 mV to the step potential +60 mV evoked a clear decrease in fluorescence of VSD-FR189-188 (normalized signal amplitude: 0.4 ± 0.04%, n = 5 cells; Fig 4A). The VSD-FR189-188 signal in response to a train stimulation with repetitive voltage steps reflected the periodic voltage changes in accordance to the train frequencies of 2.5 and 5 Hz (Fig 4B and 4C).

We also tested VSD-FR189-188 probe in HEK293 cells with a command protocol stepping from a holding potential of −110 mV to conditioning step potentials of -170 mV, -20 mV, 30 mV, and 130 mV with step duration of 100 ms (Fig 4D). For each step, the changes in fluorescence corresponded to applied voltage steps and increased accordingly to voltage step amplitude.

**Fig 5:**
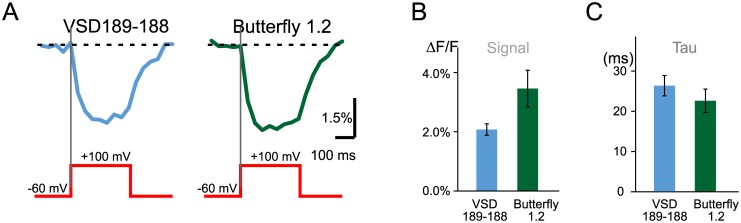
Comparison of signal amplitudes and dynamics between VSD189-188 and Butterfly 1.2 voltage-sensitive probes tested in similar conditions in HEK293 cells. (A) ON-responses evoked by voltage steps. (B) signal amplitude ±SEM measured against the baseline at the end of each voltage step. (C) Tau ±SEM, a time constant obtained from exponential fits of the ON-responses shown in A.

We next compared the VSD-FR189-188 signal to that of the previously reported voltage-sensitive probe Butterfly 1.2 by recording its ON-response with a CCD camera at 510–550 nm emission band, which is nearly optimal for single emission band recording [12]. VSD-FR189-188 signal was recorded with a 575–630 nm bandpass filter. Our experiments demonstrated that both the tau and amplitude of ON-responses in these probes were comparable (signal amplitude, VSD-FR189-188: 2.1 ± 0.3%, Butterfly 1.2: 3.5 ± 0.7%; signal rise tau, VSD-FR189-188: 26.3 ± 2.6 ms, Butterfly 1.2: 22.7 ± 3.0 ms; n = 6 cells; Fig 5).

Although the contrast of the biosensor is modest (about 0.4–3% dF/F, depending on measurement protocol and cell line), our experiments successfully demonstrated that the design of reporter domain based on VSD insertion into circularly permuted red FP can be used for voltage imaging. Moreover, on the average VSD-FR189-188 showed better performance in HEK293 cells than cpmKate-based sensors designed with fusion-to-cpFP topology.

Low contrast of VSD-FR189-188 presumably demonstrates that the length of polypeptide linker between functional domains requires further optimization. However, synchrony of changes in the fluorescent signal and membrane potential constitutes a proof of principle for our biosensor design.

Red-shifted emission makes this fluorescent tool potentially suitable for the whole body imaging. Thereby further modification and improvement of voltage biosensors based on this topology will allow us to get closer to the creation of this important tool for electrophysiology.

In this study we showed that the breakpoints suitable for permutation could be successfully applied to a split FP construction. Since permutation is technically easier than direct split FP cloning one can use such approach for a preliminary design of multiple versions of split FPs.

Here we reported the novel bimolecular fluorescence complementation (BiFC) constructs based on the monomeric red fluorescent protein. BiFC represents a common approach to protein-protein interactions research, and red emission of the fluorescent part may extend exploitation of this method to optically dense systems such as fresh tissue sections and even whole body. In model protein-protein association system presented in current work by leucine zippers pZip189-188 demonstrated a relatively fast fluorescence appearance and brightness comparable to the original protein. We suppose that new FusionRed splits are ready-to-use components applicable for the wide spectrum of molecular interactions researches.

We showed that non-covalently associated parts of cpRFP split by the voltage-sensitive domain could be used as a fluorescent core in genetically encoded voltage sensor. In fact, this functional principle is more related to fluorescence complementation than circular permutation technique. This sensor offers simple single channel detection and it is potentially suitable for whole body imaging due to appropriate emission maximum. We believe that low contrast of pVSD-FR189-188 could be further corrected by a screening through different polypeptide linkers between VSD and FP parts.

## Supporting information

S1 Fig Schematic of the concept of circular permutants engineering from a tandem FusionRed template. Key: green circle is pQE30 vector, blue circle is pEGFP-N1 vector, red arrow is the first copy FusionRed tandem, brown arrow is the second copy FusionRed tandem, magenta arrow is cpFusionRed, n and n-1 are numbers of amino acids (numbering by FusionRed) encoded by the terminal triplets of resulting cpFusionRed, thin arrows are primer annealing sites.
(EPS)

S2 Fig Schematic of split FusionRed cloning. The pair of FusionRed fragments were amplified using pQE-30 cpFR189-188 as a PCR-template. These fragments were subcloned into pAqMHalvesZip1 plasmid vector. NZ and CZ are two genes of N-terminal and C-terminal leucine zippers which were fused to the parts of FusionRed in resulting plasmid.
(EPS)

S3 Fig Schematic of VSD insertion into cpFusionRed. The construct was engineered using overlap extension PCR. The products of FusionRed fragments PCR-amplification (red lines) were annealed to the VSD fragment amplified using oligos containing FusionRed-homologous sequences (blue line with red ends). Obtained duplex was then PCR-amplified with a pair of the flanking primers. The final PCR-fragment was inserted into pEGFP-N1 vector.
(EPS)

S1 Appendix Materials and methods. (DOCX)
