# Supplementary material for: Insertion of the voltage-sensitive domain into circularly permuted red fluorescent protein as a design for genetically encoded voltage sensor
Source: PLoS One. 2017 Sep 1;12(9):e0184225. doi: 10.1371/journal.pone.0184225 (PMC5580962; doi:10.1371/journal.pone.0184225)
Supplement: S1 Appendix — (DOCX) [file pone.0184225.s004.docx]

**Supplementary materials and methods**

**Vector construction**

To generate the cpFR variants, we created a tandem fusion template of two FusionRed coding sequence copies. The coding sequence of FP gene was PCR-amplified from FusionRed-C plasmid (Evrogen) template and inserted into the pQE30 vector (Qiagen) in two copies separated by a GGTGGS polypeptide linker. This tandem construct was used as a template for circular permutants sequences amplification (full cloning scheme is shown in S1 Fig).

**cpFRs characterization in *E. coli* and *in vitro***

Permuted variants of FusionRed with N-terminal 6His tag were expressed in *Escherichia coli* XL1 Blue strain (Invitrogen) and purified using the TALON metal-affinity resin (Clontech). Absorption spectra were recorded with a Beckman DU520 UV/VIS Spectrophotometer. A Varian CaryEclipse Fluorescence Spectrophotometer was used for measuring the fluorescence excitation-emission spectra. For molar extinction coefficient determination, we relied on measuring the mature chromophore concentration. Proteins were alkali-denatured with an equal volume of 2M NaOH and absorbance spectra were measured immediately. It is known that alkali-denatured DsRed-like chromophore converts to the GFP-like one [1, 2] with an extinction coefficient 44,000 M^-1^cm^-1^ at 452 nm under these conditions. We calculated molar extinction coefficients for the native states on the basis of the absorption of the native and alkali-denatured proteins. The results are shown in Table 1.

**Cell culture and transfection**

PC12 cells were grown in RPMI1640 medium supplemented with a mixture of 5% fetal calf serum-10% horse serum (Sigma) and plated onto poly-D-lysine-coated coverslips. Transfections were carried out 24 h after plating by electroporation in Lonza nucleofector device according to the manufacturer’s instructions for PC12 electroporation. After a day of cultivation, the transfection efficiency was evaluated and NGF (Sigma) was added to induce the differentiation of PC12 cells into the neuron-like cells. Experiments with PC12 cells were carried out 48 h after induction of differentiation.

HEK293 were used for both split-zippers association detection and for the electrophysiological studies with VSD. Cells were grown in full DMEM medium on glass bottom dishes (Fluorodish). Transfection was performed using FuGene 6 transfection reagent (Promega). Fluorescent visualization was performed 48 h after transfection.

**References**

1. Gross LA, Baird GS, Hoffman RC, Baldridge KK, Tsien RY. The structure of the chromophore within DsRed, a red fluorescent protein from coral. Proc Natl Acad Sci U S A. 2000;97: 11990–5. doi:10.1073/pnas.97.22.11990

2. Baird GS, Zacharias D a, Tsien RY. Circular permutation and receptor insertion within green fluorescent proteins. Proc Natl Acad Sci U S A. 1999;96: 11241–11246. doi:10.1073/pnas.96.20.11241
